# Role of liquid biopsy for thoracic cancers immunotherapy

**DOI:** 10.37349/etat.2020.00012

**Published:** 2020-06-29

**Authors:** Raimondo Di Liello, Flora Cimmino, Soraya Simón, Emilio Francesco Giunta, Vincenzo De Falco, Paloma Martín-Martorell

**Affiliations:** 1 Medical Oncology, Department of Precision Medicine, Università degli Studi della Campania Luigi Vanvitelli, 80131 Naples, Italy; 2 Medical Oncology Department, Hospital Clínico Universitario de Valencia, 46010 Valencia, Spain; 3 CEINGE Biotecnologie Avanzate, 80131 Naples, Italy; Università degli studi della Campania, Italy

**Keywords:** Liquid biopsy, thoracic cancer, non-small cell lung cancer, small cell lung cancer, mesothelioma, circulating tumor cells, tumor mutational burden, programmed cell death ligand 1

## Abstract

Immunotherapy has shifted the therapeutic landscape in thoracic cancers. However, assessment of biomarkers for patient selection and disease monitoring remain challenging, especially considering the lack of tissue sample availability for clinical and research purposes. In this scenario, liquid biopsy (LB), defined as the study and characterization of biomarkers in body fluids, represents a useful alternative strategy. In other malignancies such as colorectal cancer, breast cancer or melanoma, the potential of LB has been more extensively explored for monitoring minimal residual disease or response to treatment, and to investigate mechanisms of resistance to targeted agents. Even if various experiences have already been published about the applications of LB in immunotherapy in thoracic cancers, the standardization of methodology and assessment of its clinical utility is still pending. In this review, the authors will focus on the applications of LB in immunotherapy in non-small cell lung cancer, small cell lung cancer, and malignant pleural mesothelioma, describing available data and future perspectives.

## Introduction

Liquid biopsy (LB) represents a noninvasive approach for the analysis of tumor-derived biomarkers in biological fluids. The main components of LB include circulating tumor cells (CTCs), cell-free molecules like circulating tumor DNA (ctDNA), circulating tumor RNA (ctRNA), and extracellular vesicles (EV) [1–3]. Analysis of CTCs and ctDNA represents nowadays the most studied application of LB. CTCs are intact tumor cells released into the bloodstream from primary or metastatic lesions, while ctDNA comprises fragments of 160–180 base pairs released into the circulation from tumor cells. In healthy individuals, the bulk of circulating cell-free DNA (cfDNA) derives from apoptotic hematopoietic cells, while in cancer patients, it includes ctDNA derived from different cell types. Specifically, ctDNA released from tumor cells differs from cfDNA from apoptotic hematopoietic cells in terms of characteristic somatic genomic alterations [4]; also, the presence of larger DNA fragments suggests a non-hematopoietic origin [5]. Quantification of cfDNA levels in cancer patients’ blood compared to healthy subjects may also have various clinical applications, especially in monitoring treatment response, predicting resistance and improving patients’ outcome [6]. The availability of LB is rapidly changing the approach of clinical management of cancer patients allowing researchers and clinicians to characterize and monitor tumor dynamics without performing invasive tissue biopsies. Different authors have already described the potential applications of LB for the detection of minimal residual disease [7], to identify prognostic and predictive factors or to assess the genomic profiling of various malignancies including colorectal cancer [8, 9] and breast cancer [10]. Considering that the main genomic alterations identified in tumor tissue (point mutations, rearrangements, amplifications or gene copy variations) can be detected also in cfDNA, this is considered, to date, the most studied and clinically meaningful component of LB, especially for the characterization of oncogene-driven tumors such as epithelial growth factor receptor mutated (EGFRmut) non-small cell lung cancer (NSCLC) [11]. A post-hoc analysis of the multicentric, open-label, randomized, phase III ENSURE study, that evaluated the efficacy and safety of erlotinib *versus* gemcitabine plus cisplatin as first-line treatment for stage IIIB/IV EGFRmut NSCLC patients [12] showed a 76.7% of agreement between *EGFR* testing for exon 19 deletion and exon 21 (L858R) mutation in plasma using the Cobas EGFR Mutation Test v2 (Roche Molecular Systems, Inc.) and standard *EGFR* testing in tissue. Based on this result, on June 1st 2016, Cobas was approved for plasma specimens as a companion diagnostic test for the detection of *EGFR* exon 19 deletions or exon 21 L858R mutation becoming the first “liquid biopsy test” officially approved by the U. S. Food and Drug Administration [13]. From this approval, further evidence about the clinical utility of LB in NSCLC has been produced [14, 15] and the positive results of the NILE (Non-invasive *versus* Invasive Lung Evaluation) study that showed a concordance > 98.2% with a 100% positive predictive value of cfDNA *versus* tissue assessment of *EGFR*, *ALK*, *ROS1* and *BRAF* status [16], have recently confirmed the value of LB for biomarkers analysis of NSCLC patients. Another promising application of LB in lung cancer regards the estimation of the risk of recurrence in early stage patients after radical treatment, especially in absence of clinical or radiological sign of disease as stated in the TRACERx study, where pre- and post-surgery ctDNA assessment correlated with disease recurrence, anticipating conventional imaging procedures [17]. Similarly, Chaudhuri et al. [18], reported that ctDNA detection in stage I-IIIA NSCLC patients could identify recurrence significantly earlier than standard radiographic assessment.

**Figure 1.**
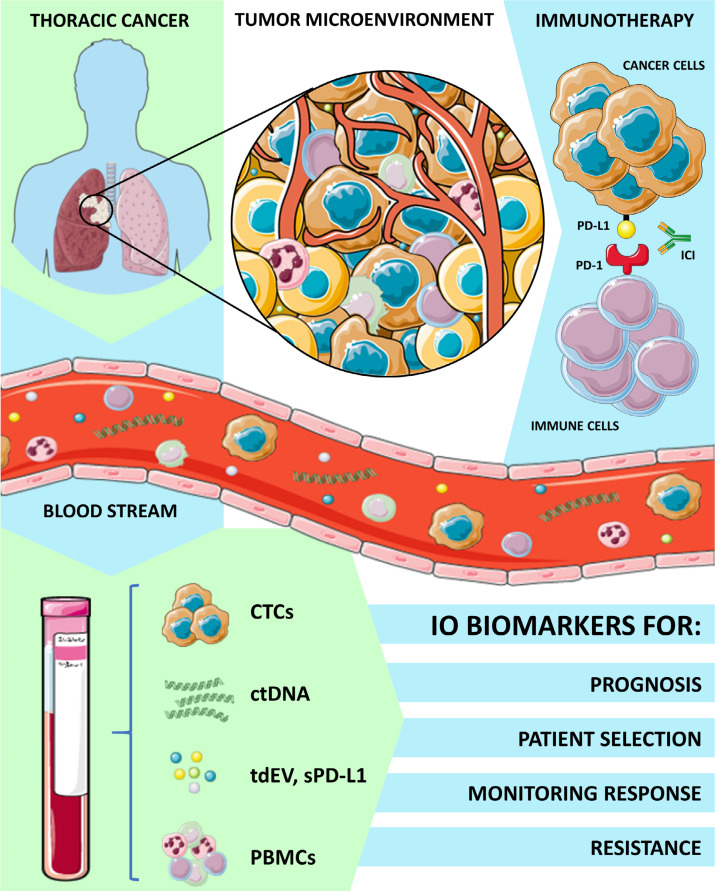
Applications of LB and circulating biomarkers analysis in thoracic cancers immunotherapy. IO: immuno-oncology

Immunotherapy has revolutionized the approach of cancer therapy in the last years leading to multiple major advantages in the treatment of different cancer types and has completely transformed the therapeutic landscape of many thoracic malignancies. The immune checkpoint inhibitors (ICIs) as monotherapy or in combination with chemotherapy have significantly improved overall survival (OS) when compared with standard treatment leading to unprecedented 5-year OS rates [19] and are now considered the standard of care in different settings [20]. The role of LB for immunotherapy biomarker assessment has been extensively explored in melanoma patients. In this setting, programmed death ligand 1 (PD-L1) expression on CTCs and ctDNA quantitative serial assessment of *BRAFV600* and *NRASQ61*/*G12*/*G13* mutations at baseline and during therapy, have been reported as predictive biomarkers of clinical benefit and response to treatment with programmed cell death-1 (PD-1) inhibitors [21, 22]. However, less experience is available in other malignancies where immunotherapy has emerged later as a valid therapeutic option. For instance, the detection of CTCs overexpressing PD-L1 and high levels of soluble PD-L1 could have a potential prognostic value in head and neck cancer [23, 24] and could guide patient selection in muscle invasive and metastatic bladder cancers [25]. Circulating biomarkers shed from the tumor microenvironment such as cytokines, and peripheral monoclonal blood cells (PBMCs) are currently used in immuno-oncology for the prediction of immune response or adverse effects. Thus, in this scenario, LB that refers to analysis of tumor-derived biomarkers into bloodstream, such as CTC, ctDNA, cfDNA, proteins, EV, is a modern tool to evaluate and monitor the complexity of patients’ response to immunotherapy, as summarized in Figure 1. In this review, we have discussed all the different experiences and applications of LB in thoracic cancer immunotherapy scenario.

## LB in thoracic cancers immunotherapy

Different biomarkers have been proposed to predict response to immunotherapy in NSCLC. PD-L1 expression, assessed on tumor cells and imm une-cells derived from biopsy specimens are associated with poor tumor differentiation an d inferior OS in the advanced setting, while discordant data are available on the prognostic value of PD-L1 in patien ts receiving (neo}adjuvant treatment for early stage disease [26, 27]. Based on available data about correlation between PD-L1 expression and higher response rate and survival in NSCLC patients treated with ICIs, PD-L1 expression is widely used both in clinical practice and trial design as biomarker of selection for patients. Nowadays, tissue-based PD-L1 expression is the only validated biomarker to guide treatment decisions [28]. The profiling of lung cancer tissue implies invasive biopsies, with risks of complications and significant delays in the starting of treatment. Furthermore, tissue-based PD-L1 assessment does not recapitulate the overall tumor heterogeneity that may explain the common discordant clinical responses of patients to immunotherapy even in presence of high PD-L1 positivity. In particular, dynamic changes in disease microenvironment and immune landscape can occur during immunotherapy imposing serial repetition of biopsies to personalize the treatment approach for resistant disease [29]. Therefore, different LB techniques have been evaluated to identify biomarkers from blood samples which may reflect this dynamism, to predict ICIs response without exposing patients to multiple invasive tissue biopsies.

## Analysis of CTCs, exosomes, and PD-L1 as early-response biomarkers

### CTCs and exosomes

CTCs have been reported as an independent prognostic factor of short OS in NSCLC [30] and the mere presence of detectable CTCs could be considered a reflection of tumor burden or invasiveness [31, 32]. Exosomes are 30–200 nm EV that carry genetic and molecular information including DNA, RNA, and proteins of their original cells, among them, tumor-derived EV (tdEV) refers to vesicles deriving from tumors that express epithelial cell adhesion molecule and cytokeratin that, in contrast to CTCs, do not have a nucleus. In lung cancer, circulating exosomes may contain tumor-related biomarkers, such as EGFR, cytokeratins and a variety of microRNAs and their detection could provide useful information for diagnosis [33]. As CTCs, tdEV can be found in NSCLC patients and are associated with worse survival [34]. Considering their prognostic role, the quantification CTCs and tdEV has been explored as possible early markers of response to ICIs. Recently, Tamminga et al. [35], reported the results of the evaluation of the predictive role of CTCs and tdEV in a large prospective series of 104 stage IIIB-IV NSCLC patients treated with ICIs who underwent blood samples analysis at baseline and 4 to 6 weeks after start of therapy. They observed detectable CTCs in one third of patients but early response was not different from those without CTCs at baseline or during treatment. Interestingly, they reported a more significant correlation (even if not statistically significant at a multivariate analysis) of CTCs count decreasing (39% *vs.* 8% in tumor response, *P* = 0.08) and a higher durable response rate in patients without CTCs detectable at baseline or during treatment (46% *vs.* 21%, *P* = 0.02 and 54% *vs.* 12%, *P* < 0.01 respectively). Fewer clinically relevant data were reported for tdEV that were not associated with either early or durable tumor response. They also confirmed the prognostic value of CTCs and tdEV showing that both were associated with worse progression free survival (PFS) and OS. Unfortunately, a low number of CTCs is usually found in a standard blood sample (most of the CTC positive samples had 1 CTC in this series) and this could limit their clinical applicability. Recently Shin et al. [36], described the potential application of circulating exosomes for diagnosis of early stage lung cancer but as tdEV, their application in lung cancer immunotherapy is still immature and needs further validation.

### PD-L1-positive [PD-L1(+)] CTCs

Considering the prognostic and predictive role of tissue-based PD-L1 in advanced NSCLC (aNSCLC), different LB techniques have been proposed to detect PD-L1 expression in blood samples in order to overcome the issues related to tissue-based evaluation and support histological analysis. For instance, the positivity of PD-L1 immunofluorescent staining on CTCs and its prognostic significance has been explored. Boffa et al. [37], reported data from the analysis of PD-L1 expression on CTCs from a prospective multi-institutional study evaluating CTC detection as a surrogate for tissue diagnosis in patients with suspected lung cancer (NCT01830426). Interestingly, the authors stated that not all the identified circulating cells in patient samples were genetically confirmed to be malignant and could at least in part represent a transition in cancer cell phenotype and a PD-L1 expressing cell population at some level of the host-tumor interface. They refer to them as circulating cells associated with malignancy (CCAMs) instead of CTCs even though single-cell sequencing revealed copy number variations consistent with a malignant origin. They reported a PD-L1(+) CCAMs in 26 of 112 treatment-naïve NSCLC patients studied (23%) compared with no detection in healthy controls. They evaluated also the relationship between PD-L1(+) CCAMs and long-term survival showing that lung cancer patients with > 1.1 PD-L1(+) CCAM/mL (*n* = 14) experienced a worse median survival and a worse 2-year survival than those with ≤ 1.1 PD-L1 (+) CCAM/mL (31.2% *vs.* 78.8%, *P* = 0.00159) and that expression of > 1.1 PD-L1(+) CCAM/mL was an independent predictor of mortality risk at multivariate analysis [hazard ratio (HR): 3.85, 95% confidence interval (CI): 1.64–9.09, *P* = 0.002]. Beyond its prognostic significance, the quantification of PD-L1(+) CTCs has been also correlated to response to treatment and clinical outcomes of aNSCLC patients treated with ICIs (Table 1).

**Table 1.** Reported studies on PD-L1(+) CTCs in NSCLC

**Authors**	**Pts (*n*)**	**Blood tubes**	**ICI**	**Outcome correlation**
Nicolazzo et al. [38]	24	CellSave^¶^	N	Better if decreasing on treatment
Guibert et al. [40]	96	*	N	Poorer if present at baseline
Kulasinghe et al. [41]	33	EDTA/Streck	N	No correlation reported
Dhar et al. [42]	22	EDTA	P	Better if > 50% at baseline

¶ CellSave preservative tubes (Janssen) containing EDTA and a cell fixative;

* not reported; pts: patients; *n*: number; N: nivolumab; P: pembrolizumab

Assessment of PD-L1(+) CTCs in NSCLC has been first used as clinical predictive marker for patients treated with the anti-PD-1 nivolumab [38]. At baseline, the number of CTCs detected from patient blood samples ranged from 1 to 20 (median number of CTCs 5.2), most of them (83%) with a high frequency of PD-L1 expression (95%). After three months of treatment, the fraction of PD-L1(+) CTCs ranged from 25% to 100%, while after six months they decreased to 50%, showing a clinical benefit in the group with PD-L1-negative CTCs in contrast with PD-L1(+) CTC group. Despite these data, even though both the presence of CTCs and the PD-L1 expression were associated with poorer outcomes, the lack of a significant number of patients with PD-L1-negative CTCs did not provide strong evidences regarding the real prognostic and predictive relevance of this marker [39].

In 2018, Guibert et al. [40], detected a median proportion of CTCs expressing PD-L1 of 17.2% in 93% of aNSCLC patients before nivolumab treatment. No correlation has been observed with PD-L1+ diagnostic tissue biopsies (72%, *P* = 0.77); this may be due to the time between tissue biopsy and pre-treatment blood collection (median time was 7.8 months with 1–12 months in 69.8% and more than one year in 24.5% of the cases). They also showed that in patients treated with PD-1 inhibitor, pretreatment PD-L1(+) CTCs were associated with poor prognosis. In contrast to these evidences, other authors reported no correlation between PD-L1(+) CTCs and clinical outcomes in patients treated with nivolumab [41].

Clinical significance of PD-L1(+) CTCs has also been investigated in patients treated with the anti-PD-1 pembrolizumab. In the study of Dhar et al. [42], the majority of patients of the series (~97%) showed ≥ 1 PD-L1(+) CTCs at baseline and, importantly, those with > 50% PD-L1(+) CTCs experienced an improved PFS under treatment.

Collectively, available results are still not definitive for giving a significant predictive value of PD-L1(+) CTCs for immunotherapy.

### Soluble PD-L1

Beyond PD-L1 detection on CTCs, soluble PD-L1 (sPD-L1) has also been proposed as an immunity-related biomarker that could be analyzed in plasma. sPD-L1 derives from an alternative splicing of PD-L1 mRNA or a proteolytic cleavage of membrane-bound PD-L1 [43, 44] and is higher among NSCLC patients in comparison to healthy subjects [45].

The detection of sPD-L1 was associated with poor prognosis of lung cancer patients by Okuma et al. [46], that analyzed sPD-L1 plasma concentration in 96 patients with NSCLC and small cell lung cancer (SCLC) treated with chemotherapy. They reported that with a cut-off of 3.357 ng/mL, OS was significantly reduced in patients with high plasma sPD-L1 levels (13.0 *vs.* 20.4 months, *P* = 0.037) showing at a multivariate analysis that high sPD-L1 levels were significantly related to poor prognosis (HR: 1.99, *P* = 0.041) reflecting a possible association with suppression of anti-tumor immunity [47]. Unfortunately, considering that plasma samples were not collected at the time of surgery, no correlation with PD-L1 tissue-based assessment was performed. The role of sPD-L1 has also been investigated in patients treated with nivolumab in a larger cohort of 43 NSCLC patients [48]. The authors reported no statistical difference in sPD-L1 plasma concentrations at the baseline between responders and non-responders to immunotherapy or between patients presenting with clinical benefit compared to those who did not. Furthermore, no correlation between sPD-L1 plasma concentrations at diagnosis and level of expression of tissue PD-L1 in immunohistochemistry (IHC) according to different cut-offs was shown. However, at first tumor evaluation during nivolumab, sPD-L1 plasma concentrations were significantly higher in non-responders with a median value of 67.64 pg/mL (46.36–75.14) compared to 32.94 pg/mL (24.89–58.91) in responders (*P* = 0.031) and median sPD-L1 plasma concentrations were significantly higher in patients without clinical benefit compared to patients with clinical benefit (*P* = 0.024). Moreover, in case of increase of sPD-L1 plasma concentrations between the starting of nivolumab and the first tumor evaluation (*n* = 12), overall response rate (ORR) was 17% (*n* = 2) *versus* 68% (*n* = 13) in case of decrease or stability of sPD-L1 plasma concentrations (*n* = 19, *P* = 0.005). Using 33.97 and 36.36 pg/mL as sPD-L1 cut-off concentrations, they classified patients in low sPD-L1 and high sPD-L1 expressors, showing a difference of 60% (*P* = 0.002) in ORR and of ~9 months in PFS (*P* = 0.041) between the two groups. In addition, patients with low sPD-L1 plasma concentration at first tumor evaluation had a median OS not reached [CI: 13.6-not reached (NR)] *versus* 6.2 months (CI: 2.4-NR) for patients with high sPD-L1 concentrations at first tumor evaluation (*P* = 0.087).

These results imply that CTCs, PD-L1(+) CTCs and sPD-L1 might have a role in monitoring ICIs response in aNSCLC, however, further studies–mainly prospective and with a larger sample size–are required to standardize detection and characterization of these biomarkers, to avoid misinterpretations and support future clinical applications.

## Analysis of ctDNA profiling to estimate TMB

Tumor mutational burden (TMB) is defined as the total number of somatic mutations per coding area of a tumor genome and is commonly expressed as mutations per megabase (mut/Mb) [49]. The presence of a high number of somatic mutations is correlated with the production of modified proteins that can represent tumor-specific neoantigens capable of activating anti-tumor immune responses [50]. Therefore, TMB measured by whole exome sequencing or next generation sequencing has been used as a surrogate of the tumoral neoantigen load, introducing the rationale for its use as an immunotherapy efficacy biomarker [51, 52]. LB is becoming a common alternative to tumor tissue samples to assess TMB using ctDNA despite the lack of standardization that leads to difficult interpretation of the results. To overcome this issue, different international projects are ongoing to harmonize this type of analysis [53].

TMB based on ctDNA or [blood TMB (bTMB)] was assessed in different clinical trials of immunotherapy in NSCLC and SCLC [54] (Table 2).

**Table 2.** Analysis of bTMB in lung cancer clinical trials

**Authors**	**Disease**	**Trial**	**Treatment**	**Cut-offs^¶^**
Socinski et al. [55]	NSCLC	B-F1RST	A	14.5, 16
Gandara et al. [56]	NSCLC	POPLAR/OAK	A	10, 16, 20
Rizvi et al. [57]	NSCLC	MYSTIC	D/T	20
*[58]	NSCLC	NEPTUNE	D/T + CT	20
Horn et al. [63]	SCLC	IMpower133	A + CT	10, 16

* data not published;

¶ mut/Mb; A: atezolizumab; D: durvalumab; T: tremelimumab; CT: chemotherapy

The B-F1RST trial (intention-to-treat (ITT) population = 152) was the first prospective study to evaluate bTMB as a biomarker to predict benefit of first line atezolizumab monotherapy. bTMB high (≥ 16 mut/Mb; ≥ 14.5 mut/Mb) predicted better ORR with immunotherapy *versus* bTMB low (< 16; 28.6% *vs.* 4.4%) in the biomarker-evaluable population (BEP). In bTMB ≥ 16 mut/Mb *vs.* < 16, median PFS was 5.0 *vs.* 3.5 months and median OS was 23.9 *vs.* 13.4 months [55]. Unfortunately, no comparison between bTMB and TMB evaluated on tissue samples or [tissue TMB (tTMB)] was performed. In 2018, Gandara et al. [56], reported retrospective data from the study of bTMB in patients treated with atezolizumab in clinical trials. In the phase II POPLAR study of atezolizumab *versus* chemotherapy in second line aNSCLC, increased bTMB was associated with improved benefit in terms of PFS and OS in favor of atezolizumab. Considering that the stronger PFS benefit was observed at the cut-off of ≥ 16 (HR: 0.57, 95% CI: 0.33–0.99), it was selected for confirmatory analysis in the phase III OAK study of atezolizumab *versus* docetaxel in patients with previously treated aNSCLC. Patients with bTMB ≥ 16 obtained significant PFS benefit (HR: 0.65, 95% CI: 0.47–0.92 *P* = 0.013) from atezolizumab *versus* docetaxel and a median OS of 13.5 months in those treated with atezolizumab *versus* 6.8 months with docetaxel was demonstrated in this same bTMB population (HR: 0.62, 95% CI: 0.43–0.9).

The association between bTMB and clinical outcomes was also evaluated in the phase III MYSTIC trial of durvalumab with or without tremelimumab as first-line treatment of aNSCLC. The trial missed its primary end point of OS in PD-L1-selected patients, however, a retrospective exploratory analysis of 72.4% of plasma specimen of the ITT population was performed. In patients with bTMB ≥ 20 mut/Mb, the median OS was 21.9 months (CI: 11.4–32.8) for patients treated with durvalumab plus tremelimumab, 12.6 (CI: 7.6–18.6) with durvalumab alone, and 10.0 (CI: 8.1–11.7) with chemotherapy [57]. This concept led to the incorporation of bTMB as a prospective endpoint of the NEPTUNE study comparing the combination of durvalumab plus tremelimumab with chemotherapy for patients with treatment-naïve aNSCLC. The primary endpoint of the study was OS in patients with bTMB ≥ 20 mut/Mb and, even if the official results are not yet available, a press release from AstraZeneca in August 2019 confirmed the negative results in the pre-specified biomarker-driven population [58]. Overall, in bTMB studies in NSCLC, the correlation between bTMB and tTMB has been reported as positive but generally modest: Spearman’s correlation coefficient was 0.64 (95% CI: 0.56–0.71) in POPLAR/OAK and 0.6 (no CI given) in MYSTIC. Despite the imperfect correlation, these results suggest that increased bTMB and increased tTMB are both similarly associated with improved benefit of immunotherapy [59]. Unlike NSCLC, the role of TMB in SCLC patients treated with immunotherapy is still unclear. Despite SCLC being characterized by a high somatic mutational burden [60], due to the strong association with smoking habit [61, 62], the identification of predictors of SCLC response to ICIs remains challenging and the use of LB has been reported only in the phase III IMpower 133 trial of atezolizumab plus chemotherapy in patients with extensive-stage, treatment naïve SCLC [63]. Exploratory correlative bTMB study on 351 patients of the BEP group showed no correlation between bTMB and clinical benefit maybe due to the high myelosuppressive activity of the backbone chemotherapy, as speculated by the authors. Moreover, considering the difficulty to obtain biopsy specimen, tTMB analysis has not been performed in the same cohort and no correlation between bTMB and tTMB was reported. Nevertheless, these data are discordant from previously reported results of the impact of tTMB in the phase I/II trial CheckMate 032 of nivolumab alone and nivolumab plus ipilimumab in recurrent SCLC [64]. In this study, 211 (53%) of all 401 treated patients were evaluable for tTMB-based efficacy analysis and were divided into tertiles by the total number of somatic missense mutations (low, 0 to < 143 mutations; medium, 143 to 247 mutations; and high, ≥ 248 mutations). Within both the nivolumab monotherapy and combination arm, a positive correlation between the ORR, PFS and OS and TMB was reported [65]. These data support the evidence that LB would help to improve the amount of data for TMB in lung cancers, where tumor biopsies are rarely available.

## Analysis of ctDNA levels to monitor disease evolution

The level of cfDNA and its integrity, defined as the ratio of long-base pair (bp) cfDNA/short-bp cfDNA, have been shown to be promising diagnostic and prognostic biomarkers in other cancers like colorectal, prostate, breast and gynecological cancers [66–69]. Kitahara et al. [70], reported the first study exploring integrity of cfDNA analysis in immunotherapy, studying a cohort of metastatic colorectal cancer. They investigated whether it could be considered a response biomarker to combination of immunotherapy and chemotherapy. They conducted a non-randomized, phase II trial, HLA-A status guided, double-blinded study using a cocktail of five therapeutic epitope peptides in addition to oxaliplatin-containing chemotherapy in 96 patients. They categorized the patients into two groups according to the integrity of cfDNA (cfDNA integrity value higher or lower than the median) founding that a low cfDNA integrity value was a prognostic marker for a longer PFS with the experimental treatment (*P* = 0.027). In contrast, no significant difference in OS was reported. In thoracic malignancies cfDNA concentration measurement has been evaluated for diagnostic utility in patients with chronic obstructive pulmonary disease or for differential diagnosis of pulmonary nodules [71–73] but has never been extensively studied in advanced settings. In contrast, more data are available on the use of ctDNA levels in thoracic cancers. Based on the fact that genomic alterations of ctDNA reflect the genetic landscape of the tumor, several studies reported that ctDNA quantification levels, obtained by analysis of hotspot genetic alterations, can have a prognostic [50] and predictive role as novel biomarker in NSCLC patients treated with ICIs. Furthermore, considering that the half-life of cfDNA/ctDNA is approximately 1.5 h [24], monitoring ctDNA levels in cancer patients could be helpful to monitor the dynamic clonal selection/evolution induced by ICIs and to detect early responsiveness or resistance [74].

Iijima et al. [75], analyzed ctDNA levels in 14 NSCLC patients treated with nivolumab, of which six patients were defined as responders and eight as non-responders, based on immune response evaluation criteria in solid tumors (iRECIST). They reported a statistically significant correlation between tumor volume calculated per RECIST 1.1 and ctDNA level (*P* = 0.02) and examined the correlations between early and serial changes of ctDNA and immunotherapy efficacy. ctDNA of non-responders showed consistently high allele fraction (AF) of cancer-associated somatic mutations after treatment compared with responders that showed a rapid AF decrease, mostly within 2 weeks. Moreover, two-week changes of AF of specific cancer representative mutations, (chosen as the ones with higher baseline AF) showed complete concordance with response.

Furthermore, longitudinal changes in ctDNA levels were compared with radiographic response and survival outcomes in 28 metastatic NSCLC patients receiving ICIs by Goldberg et al. [76]. A ctDNA response was defined as a > 50% decrease in mutant AF from baseline, with a second confirmatory measurement. They found a strong correlation between ctDNA response and best radiographic response and observed also a more rapid response assessment by ctDNA than by imaging with a median time to initial response of 24.5 days by ctDNA *vs.* 72.5 days by imaging. Moreover, ctDNA response was also associated with a longer time on treatment with a median of 205.5 days in ctDNA responders *vs.* 69 in non-responders, *P* < 0.001), a lower risk of disease progression or death (HR: 0.29, 95% CI: 0.09–0.89, *P* = 0.03) and also an OS gain (HR: 0.17, 95% CI: 0.05–0.62, *P* = 0.007).

All these small-sized studies confirmed the already stated results of the B-F1RST trial: Kim et al. [77], reported that patients with insufficient ctDNA to assess bTMB had an ORR gain compared with patient with a higher ctDNA detectable [34.5% in patients with maximum somatic allelic fraction (MSAF) < 1% *vs.* 10.1% in those with MSAF ≥ 1%]. ctDNA detectability also correlated with tumor burden (number of target lesions and sum of largest diameters), a known negative prognostic factor.

Immunotherapy is characterized by potential durable benefit in aNSCLC but even among patients with initial response to ICIs, a substantial fraction ultimately progress [78]. ctDNA monitoring has been explored to detect patients with metastatic NSCLC treated with anti-programmed death 1 (ligand) [PD-(L)1] more at risk of progression or with potential long-term benefit. Hellman et al. [79], identified a cohort of 31 patients with aNSCLC characterized by a sustained clinical benefit from PD-(L)1 blockade (PFS ≥ 12 months) at a long-term follow-up of median 38.7 months (range: 14.3–81.7) with a median time of treatment of 20.4 months (range: 1.7–48.1). At a surveillance timepoint, ctDNA was not detected in 27 patients (2 of whom later radiologically progressed) and 4 patients had detectable ctDNA, all of whom ultimately progressed. Thus, detection of ctDNA during surveillance of patients with extended responses to PD-(L)1 blockade could correlate with the risk of recurrence and anticipate radiological progression. Moreover, the authors found undetectable ctDNA levels in 19 patients with long-term clinical benefit despite persistently measurable disease by imaging, in the subgroup of patients (60%) that showed partial radiological responses and underwent resection of residual disease and in the single patient that obtained a complete pathologic response (for 19.0 months). They conclude that ctDNA can be a novel marker for monitoring active residual disease showing an additional potential application of ctDNA that could provide insights to treatment planning also in patients with a long-term benefit from immunotherapy, when it is challenging to determine if discontinuing the treatment. Another exploratory application of ctDNA in ICIs efficacy monitoring is the differential diagnosis between pseudo-progression and real progressive disease (PD). Pseudo-progression is an unconventional response pattern that can occur in essentially all tumors treated with immunotherapy and is defined as an increase in the size of the primary tumor or the appearance of a new lesion followed by a decrease in tumor burden [80]. Different study proposed the use of ctDNA to diagnose pseudo-progression in melanoma patients receiving ICIs [81, 82] but only few data are available for thoracic malignancies. In a previously published case report, the study of ctDNA levels allowed the differentiation between pseudo-progression and real PD. The authors described a rapid decrease of KRAS-mutated ctDNA from two patients with KRAS-mutated lung adenocarcinoma who experienced pseudo-progression in comparison with an increase of ctDNA in a patient with true progression [83].

## Other applications on blood samples to investigate immune response

Blood samples can also be used to investigate levels of other serum biomarkers of interest, like cytokines. Sanmamed et al. [84, 85], evaluated the relationship between changes in the serum interleukin-8 (IL-8) levels, an immunomodulating chemokine produced also by tumor cells [86], and the response to immunotherapy in melanoma and NSCLC patients. They reported a positive association between the decrease of serum IL-8 level and pseudo-progression in patients with a radiological disease progression and, in addition, early decreases in serum IL-8 levels were associated with longer OS. Other authors have reported the association of tumor regression to a specific response of CD8+ T cells to neoantigens [87]. Therefore, the use of activated CD8+ T cells or other PBMCs, isolated and expanded from patients’ peripheral blood, could provide useful information on tumor microenvironment and immune response to cancer. In this scenario, the study of circulating biomarkers as IL-8 levels and PBMCs coupled with LB approach may be used to explore immune response and immune-related adverse events. [88, 89]. Overall, a potential association could be found between pseudo-progression, serum biomarkers and decreased ctDNA levels. Thus, analysis of circulating biomarkers and LB could be incorporated in the diagnostic algorithm of pseudo-progression together with histopathologic examination of enlarged or new lesions, radiologic follow-up and clinical features [90].

While ICIs represent a standard therapeutic modality in NSCLC, outcomes in malignant pleural mesothelioma (MPM) have been less positive and may be influenced by the complex structure of the tumor microenvironment [91]. In this setting LB and circulating biomarker assessment (using blood specimens but also e.g., pleural effusion samples) has been extensively explored for pathogenesis study, diagnosis and prognostic stratification focusing not only on ctDNA but, more frequently, on circulating proteins, circulating microRNAs or inflammatory and angiogenic factors [92]. Thus, to the best of our knowledge, no data on LB in MPM patients treated with immunotherapy has been published. Translational studies from ongoing clinical trials of ICIs in MPM patients as the IND-227 study (NCT02784171) of pembrolizumab plus chemotherapy in first line setting are awaited.

## Conclusion

Considering the number of standard assays needed for patient characterization and selection (e.g., IHC for diagnosis, testing for driver mutations or PD-L1 expression), in the majority of patients affected by thoracic malignancies, tissue collection remains a relevant issue, especially for NSCLC and SCLC. Immunotherapy, alone or in combination with other drugs, is now considered a standard of care for multiple indications in lung cancers, from the locally advanced to the metastatic settings. However, well established prognostic and predictive factors and biomarkers of resistance are needed. Therefore, the minimally invasive approach of LB provides a promising tool to enhance immuno-oncology research and to allow clinicians to better select patients that can benefit from immunotherapy. Giving the emerging role of ICIs in treatment of all thoracic cancers, expanding and validating the applicability of LB are needed not only in NSCLC but also in SCLC and MPM. As previously stated, tissue-based PD-L1 assessment has been characterized by validation and technical problems related to the widespread of platform and analytic method developed during time [93]. More complex issues have characterized tTMB and no consensus has been reached either about its determination, validation and clinical utility or its real predictive or prognostic value [94, 95]. bTMB and ctDNA assessment have been already studied as the first widespread application of LB in clinical trials for NSCLC but additional investigations are needed to explore their clinical utility in other thoracic cancers and in clinical practice, as well as of other circulating biomarkers such as CTCs, sPD-L1, tdEV, cytokines or PBMCs. However, we foresee some disadvantages that can slow the development of a daily practice application of LB. Indeed, the obstacles that have characterized the development of tissue-based biomarkers in immunotherapy are even more pronounced when applied to a blood-based research. As previously said, available data differ about timing of samples and platforms used for analysis, leading to a difficult comparison between data from different clinical trials or retrospective series. Moreover, apart some recent published studies, many of the previous studies focused on small number of patients or post-hoc analysis of clinical trials not designed specifically to investigate LB applications. Thus, considering that even some the most recent studies were not powered to correlate blood-based biomarkers with the “classical” tissue-based assessment, a better integration of these two approaches and the contemporary analysis of the same biomarkers with the two methods (e.g., PD-L1, TMB) are needed to overcome biopsy dependency and to shift the majority of resources on a tissue-sparing biomarker development. Further prospective, well-sized translational research projects and the systematic addition of ctDNA and bTMB monitoring in clinical trials could facilitate the assessment of the value of LB in immunotherapy, accelerating its application in thoracic cancer immuno-oncology and clinical practice.

## Abbreviations

AF: allele fraction
aNSCLC: advanced non-small cell lung cancer
BEP: biomarker-evaluable population
bp: base pair
bTMB: blood TMB
CCAMs: circulating cells associated with malignancy
cfDNA: cell-free DNA
CI: confidence interval
CTCs: circulating tumor cells
ctDNA: circulating tumor DNA
EGFRmut: epithelial growth factor receptor mutated
EV: extracellular vesicles
HR: hazard ratio
ICIs: immune checkpoint inhibitors
IHC: immunohistochemistry
IL-8: interleukin-8
ITT: intention-to-treat
LB: liquid biopsy
mCRC: metastatic colorectal cancer
MPM: malignant pleural mesothelioma
MSAF: maximum somatic allelic fraction
mut/Mb: mutations per megabase
NR: not reached
NSCLC: non-small cell lung cancer
ORR: overall response rate
OS: overall survival
PBMCs: peripheral monoclonal blood cells
PD-(L)1: programmed death 1 (ligand)
PD: progressive disease
PD-1: programmed death-1
PD-L1(+): PD-L1-positive
PD-L1: programmed death ligand-1
PFS: progression free survival
SCLC: small-cell lung cancer
sPD-1: soluble programmed death ligand 1
tdEV: tumor-derived extracellular vescicles
TMB: tumor mutation burden
tTMB: tissue TMB
WES: whole exome sequencing

## Declarations

### Copyright

© The Author(s) 2020.
